# Unveiling the Physical and Nutritional Profiling of Grains in Maize Landraces and Hybrids

**DOI:** 10.3390/foods15162933

**Published:** 2026-08-21

**Authors:** Aldo Rosales, Juan Burgueño, Valeria Gómez-Pérez, Aide Molina, Luisa Cabrera-Soto, Alberto Chassaigne, Natalia Palacios-Rojas

**Affiliations:** 1 International Maize and Wheat Improvement Center (CIMMYT), Texcoco C.P. 56230, Edo. de Mexico, Mexico; a.rosales@cgiar.org (A.R.); j.burgeno@cgiar.org (J.B.); shey_jc_kayra@hotmail.com (V.G.-P.); a.l.molina@cgiar.org (A.M.); l.cabrera@cgiar.org (L.C.-S.); a.chassaigne@cgiar.org (A.C.); 2 Agronomy Department, Universidad Autónoma de Sinaloa, Culiacán C.P. 80020, Sinaloa, Mexico

**Keywords:** genetic diversity, end-use quality, whole kernels

## Abstract

Maize (*Zea mays* L.) landraces are rich sources of genetic and nutritional diversity, yet their compositional traits remain undercharacterized compared to modern hybrids. This study compiled and analyzed data from 1554 maize samples—995 landraces and 559 hybrids—evaluated at the CIMMYT Maize Quality Laboratory over more than a decade. Traits assessed included kernel structure, macronutrients, iron, zinc, carotenoids, and anthocyanins. Landraces showed greater variability and higher average concentrations of protein (10.88 vs. 9.92%), iron (17.63 vs. 13.91 mg kg^−1^), zinc (22.70 vs. 18.98 mg kg^−1^), and anthocyanins, whereas hybrids, particularly those developed through provitamin A biofortification programs, exhibited higher average provitamin A (3.33 vs. 1.84 µg g^−1^) and total carotenoid concentrations. Correlations between kernel structural components and nutrient composition highlighted the structural basis of grain quality variation. These findings demonstrate that landraces and hybrids provide complementary nutritional profiles that can jointly support breeding, food innovation, and sustainable food systems.

## 1. Introduction

Maize (*Zea mays* L.) is a staple food for millions worldwide, particularly in regions with significant long-lasting cultural importance, such as Mexico, Mesoamerica, and parts of Latin America. This versatile crop is not only a vital source of nutrients for human consumption, but it also plays a crucial role in the socio-cultural identity of these communities. Beyond its dietary significance, maize serves diverse functions, including animal feed, biofuels, and industrial applications [1,2,3].

Despite the substantial genetic diversity in both maize landraces and improved varieties, their potential to enhance grain quality remains largely unexplored [2,4,5,6]. Previous research has concentrated on leveraging genetic diversity to improve yield traits, disease resistance, and adaptation to specific agroecological conditions [7,8,9]. However, there is a growing need to understand and utilize grain quality traits precisely, nutritional composition, processing attributes, and end-use functionality [10].

The extensive genetic variability within maize provides opportunities for a wide range of applications; traits such as kernel size, color, hardness, and composition significantly impact its end-use quality, influencing food processing techniques, feed formulations, and industrial applications [11,12]. A comprehensive understanding of these grain traits is essential for the informed selection of maize varieties tailored to specific applications.

Maize can be categorized into two primary groups: landraces and improved (open pollinated varieties and hybrids). Landraces are genetically diverse, locally adapted varieties cultivated over generations by smallholder farmers through traditional agricultural practices. These varieties exhibit unique traits and adaptations to their environments, and they serve as invaluable genetic resources for enriching elite maize germplasm and enhancing resilience to environmental changes and pathogens [5,13,14,15,16]. In contrast, hybrids result from controlled crosses between distinct parental inbred lines, yielding improved agronomic traits and uniformity, leading to widespread adoption [7].

Despite the recognized importance of investigating the end-use quality of maize, existing research faces limitations, including a narrow focus on specific grain traits and a limited number of analyzed samples [17,18,19,20,21,22,23,24,25]. Moreover, comparing samples from diverse production environments, such as freshly harvested versus stored maize, complicates analyses due to variability from different storage conditions and handling practices [26,27,28]. Addressing these limitations is crucial for advancing our understanding of maize grain quality and the implications of genetic diversity.

This study addresses these challenges by integrating one of the largest standardized datasets currently available on maize grain quality. By compiling 15,514 phenotypic observations across physical, chemical, and nutritional grain quality traits from 1554 maize landraces and hybrids analyzed under standardized laboratory protocols over more than a decade, the dataset provides a unique opportunity to document large-scale patterns of physical, compositional, and nutritional diversity that would be difficult to capture in a single controlled experiment. Although historical datasets inevitably include environmental heterogeneity, they complement traditional genotype × environment studies by revealing the breadth of diversity currently available for breeding, food innovation, and germplasm utilization. Furthermore, germplasm collections represent a unique repository of nutritional and functional diversity. While a subset of accessions has already been characterized for key grain quality traits, a large proportion of the diversity conserved in gene banks remains underexplored, representing an important opportunity for future food, nutrition, and crop improvement research. The objective is to characterize the phenotypic variability of maize kernels in terms of their physical structure, proximate composition, and nutritional content. Specifically, we aim to (i) compare key traits across landraces and hybrids, (ii) identify structural features associated with nutritional quality, and (iii) assess the potential of maize kernels, particularly those from landraces, as nutritious, whole-kernel food sources. In doing so, we highlight the value of maize genetic diversity for both food innovation and breeding strategies.

## 2. Materials and Methods

### 2.1. Grain Samples

The dataset comprised 1554 maize entries (995 landraces and 559 hybrids) analyzed at the CIMMYT Maize Quality Laboratory “Evangelina Villegas” between 2011 and 2022. Samples originated from multiple research projects, breeding programs, germplasm regeneration activities, and farmers’ field collections representing diverse agroecological regions, primarily from Latin America. Consequently, the dataset captures a broad range of environmental conditions and genetic backgrounds rather than a single controlled experiment. Although different validated analytical platforms were used over time as laboratory capacity evolved, each method was standardized and routinely implemented under CIMMYT laboratory quality assurance procedures. Previous validation studies demonstrated high agreement among methods, allowing the integration of historical data for exploratory analyses.

### 2.2. Physical, Chemical and Nutritional Analysis

Physical traits data included thousand kernel weight (TKW) (g), hectoliter weight (HW) (kg hL^−1^), percentage of the different kernel structures (tip cap, pericarp, germ, floury and vitreous endosperm), color, grain size, and grain hardness. Chemical traits included percentage of protein, starch, and oil; and nutritional traits refer to essential amino acids, percentage of lysine (Lys) and tryptophan (Trp); minerals, iron (Fe) and zinc (Zn) (mg kg^−1^); antioxidants, carotenoids in yellow maize (lutein, zeaxanthin, β-cryptoxanthin, β-carotene, 9-cis-β-carotene, 13-cis-β-carotene, provitamin A carotenoids, µg g^−1^) and total anthocyanins (µg Pel g^−1^) in blue maize.

**Figure 1:**
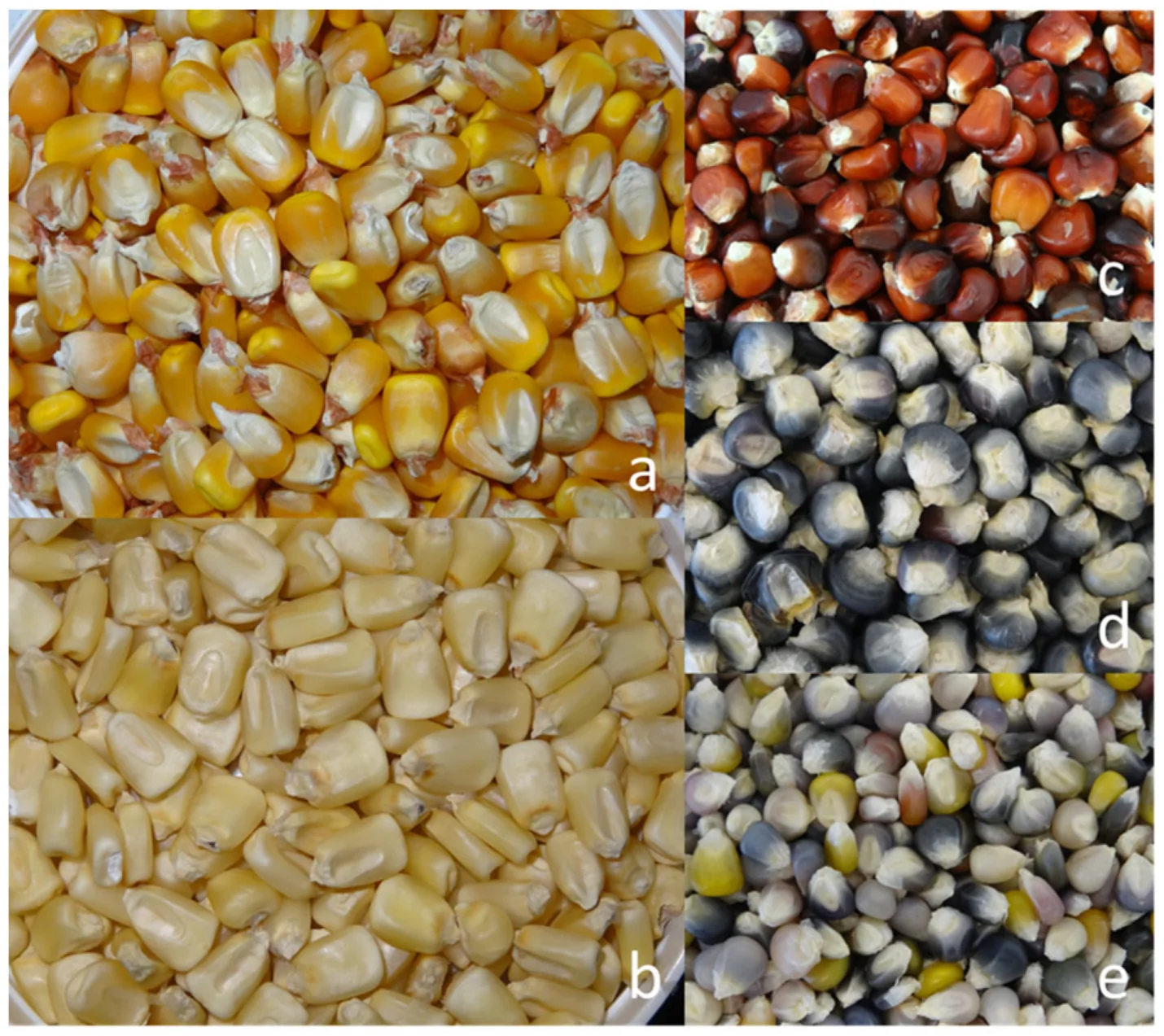
Grain color scale. (**a**) Yellow hybrid maize. (**b**) White hybrid maize. (**c**) Red landrace maize. (**d**) Blue landrace maize. (**e**) Mixed landrace maize. Representative examples of the five grain color categories used in this study. Images are intended to illustrate color classification only and do not represent kernel size.

TKW, an indirect measure of kernel size, was determined by weighing 100 kernels and multiplying the obtained weight by 10. HW, a measure of grain density, was determined using a hectoliter weight balance. The grain dissection technique was employed to assess kernel composition percentage. Preliminary grain color determination was conducted using a color scale (Figure 1, Table 1). Minolta CR-410 colorimeter was employed to obtain luminosity (L), a, and b values, providing a more precise quantitative measurement of grain color within each color category [u8].

The grain size was determined by distributing the grains through sieves of different sizes and subsequently classifying them using a size scale. The assessment of grain hardness was performed using the flotation index method, and a classification was assigned according to hardness (Table 1). Nitrogen content was determined using the Technicon Autoanalyzer II^TM^ (SEAL Analytical, Hamburg, Germany) Kjeldahl method, and the protein was estimated using the conversion factor 6.25. Total starch was calculated using a modified Megazyme method. Oil was extracted for one hour in a Soxtec^TM^ (FOSS Analytics, Hillerod, Demark) extractor using petroleum ether. All results are expressed on a dry basis.

Total carotenoids in yellow maize were determined using ultra-performance liquid chromatography (UPLC), spectrophotometry, or near-infrared reflectance spectroscopy (NIRS). UPLC analyzed provitamin A carotenoids. Iron and zinc concentrations were analyzed by inductively coupled plasma (ICP-OES) or X-ray fluorescence (XRF).

Total anthocyanins in blue maize were determined using a colorimetric method, using acidified methanol to extract anthocyanins; a higher intensity of red coloration means a higher concentration of these metabolites.

Unless specified otherwise, all procedures used in the analysis are described in detail by [29,30,31,32,33,34].

### 2.3. Statistical Analysis

Data from samples with the same genealogy were averaged for both hybrids and landraces. Therefore, 559 data points were used for hybrids and 995 data points for landraces in the statistical analyses. Because the samples originated from different environments and years, environmental effects cannot be separated from genetic effects. Therefore, the statistical analyses were designed to describe patterns of variation among groups rather than to estimate genotype, environment or genotype × environment variance components.

The unequal number of observations across traits and the lack of complete overlap among variables limited the application of more complex multivariate and mixed-model approaches. Consequently, the number of samples available varied among traits and is reported in Table 2. Hybrid samples were not analyzed for tryptophan (Trp) and lysine (Lys), while hectoliter weight (HW) was not recorded for landraces (Table 2).

All results were statistically analyzed using SigmaPlot version 15.0 (Systat Software, Inc., San Jose, CA, USA) and R software, R.4.4.x (R Foundation for statistical computing, R core team, Vienna, Austria). One-way ANOVA was used exclusively to compare predefined sample groups (landraces versus hybrids and grain color categories). Because the dataset integrates historical observations from different years and environments, the analysis was not intended to estimate genotype, environment, or genotype × environment effects.

## 3. Results and Discussion

Table 2 presents comprehensive data for all assessed traits, while Supplementary Table S1 summarizes the significant correlations between traits.

### 3.1. Grain Physical Traits

**Figure 2:**
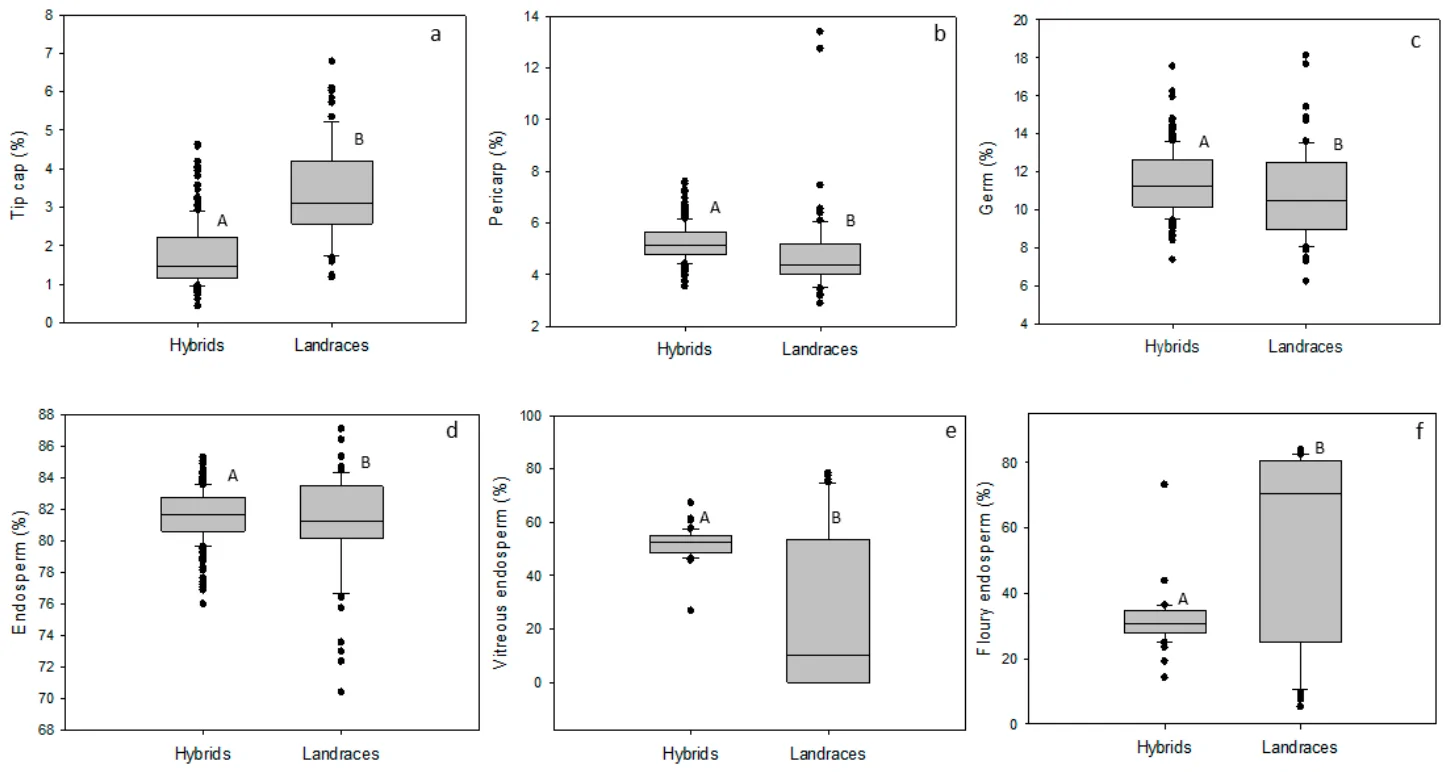
Distribution of kernel structures in hybrids and landraces: (**a**) tip cap (%), (**b**) pericarp (%), (**c**) germ (%), (**d**) endosperm (%), (**e**) vitreous endosperm (%), and (**f**) floury endosperm (%). Different uppercase letters indicate statistically significant differences among groups according to ANOVA (*p* < 0.001).

Most physical traits were analyzed using sample sizes exceeding 200, except for hectoliter weight (HW) and kernel structure percentages. Overall, landraces exhibited greater variability in physical traits than hybrids (Figure 2).

The L value describes kernel lightness (0 = black, 100 = white), while a and b values indicate color direction from green to red and from blue to yellow, respectively. Together, these parameters provide an objective characterization of grain color and visual appearance. In terms of grain color and luminosity, hybrids showed slightly higher average L values, reflecting the predominance of white and yellow kernels, whereas landraces encompassed a wider range of colors, including red and blue grains, which typically exhibit lower L values, often around 50.

Despite this diversity, grain color variation has been largely overlooked in breeding programs and commercial markets. Cabrera-Soto et al. (2023) [35] report that the Mexican maize market remains dominated by white and yellow varieties, a pattern also observed globally. This contrasts with the strong cultural relevance of blue and other pigmented maize, which are integral to traditional food systems and are commonly used in preparations associated with social and religious events [2].

Significant correlations were observed between color parameters and nutritional traits (Table S1). The a value was positively correlated with total carotenoids (R = 0.538), β-cryptoxanthin (R = 0.425), and zeaxanthin (R = 0.516), while the b value was negatively correlated with total anthocyanins (R = −0.483). These relationships have been widely exploited for high-throughput phenotyping using colorimeters or near-infrared reflectance spectroscopy (NIRS) in cereals and vegetables [30,33,36]. Additional significant correlations were detected between L value and tryptophan (R = 0.433), and between b value and oil content (R = −0.418), tryptophan (R = 0.498), and total carotenoids (R = 0.538), consistent with previous reports [37,38].

Color parameters were also associated with kernel texture. Quality protein maize (QPM) kernels are typically soft, floury, and opaque, whereas conventional maize kernels are hard, vitreous, and translucent [38]. Accordingly, L and b values reflect the higher whiteness and floury texture characteristic of QPM grains. Positive b values indicate yellow pigmentation, while negative values correspond to blue hues, which helps explain their association with anthocyanins.

Recent efforts to cultivate and breed blue and red maize have gained momentum [6,15,23,39]. In regions where maize holds strong cultural and genetic significance, such as Mexico, pigmented maize plays a central role in traditional foods, including tortillas and other nixtamalized products, which often command higher market prices than those made from white maize. Similarly, in Central America, some countries of South America, including Peru and Bolivia, pigmented maize—largely derived from landraces—remains central to culturally specific food systems and benefits from enhanced adaptation to local environments under low-input conditions.

Despite the lower number of data points available for kernel structure traits in landraces compared to hybrids, landraces exhibited greater variability in the proportion of several kernel components. Specifically, tip cap and pericarp percentages showed wider ranges in landraces than in hybrids, while germ content also displayed higher variability, although hybrids presented a slightly higher average germ proportion. The endosperm, which accounts for approximately 80–85% of the kernel dry weight [40], showed small but significant differences in average proportion between groups (Table 2; Figure 2b).

Strong negative correlations were observed between germ and endosperm (R = −0.761), as well as between pericarp and endosperm (R = −0.455), indicating a trade-off among kernel structural components. These relationships are closely linked to oil content, as discussed in the macronutrient section. Additionally, hybrids exhibited a higher proportion of vitreous (hard) endosperm compared to floury (soft) endosperm (Figure 2e,f), a distinction known to influence starch digestibility, kernel texture, and overall grain quality.

The diversity observed in kernel size, hardness, and structural composition among landraces reflects their broad genetic background, which has been progressively narrowed through breeding practices favoring uniformity to meet industrial and post-harvest processing requirements. However, traits such as tip cap and pericarp proportion may regain relevance as interest in whole-grain consumption increases, given that these structures contain a substantial fraction of kernel fiber and contribute to the nutritional value of maize.

**Figure 3:**
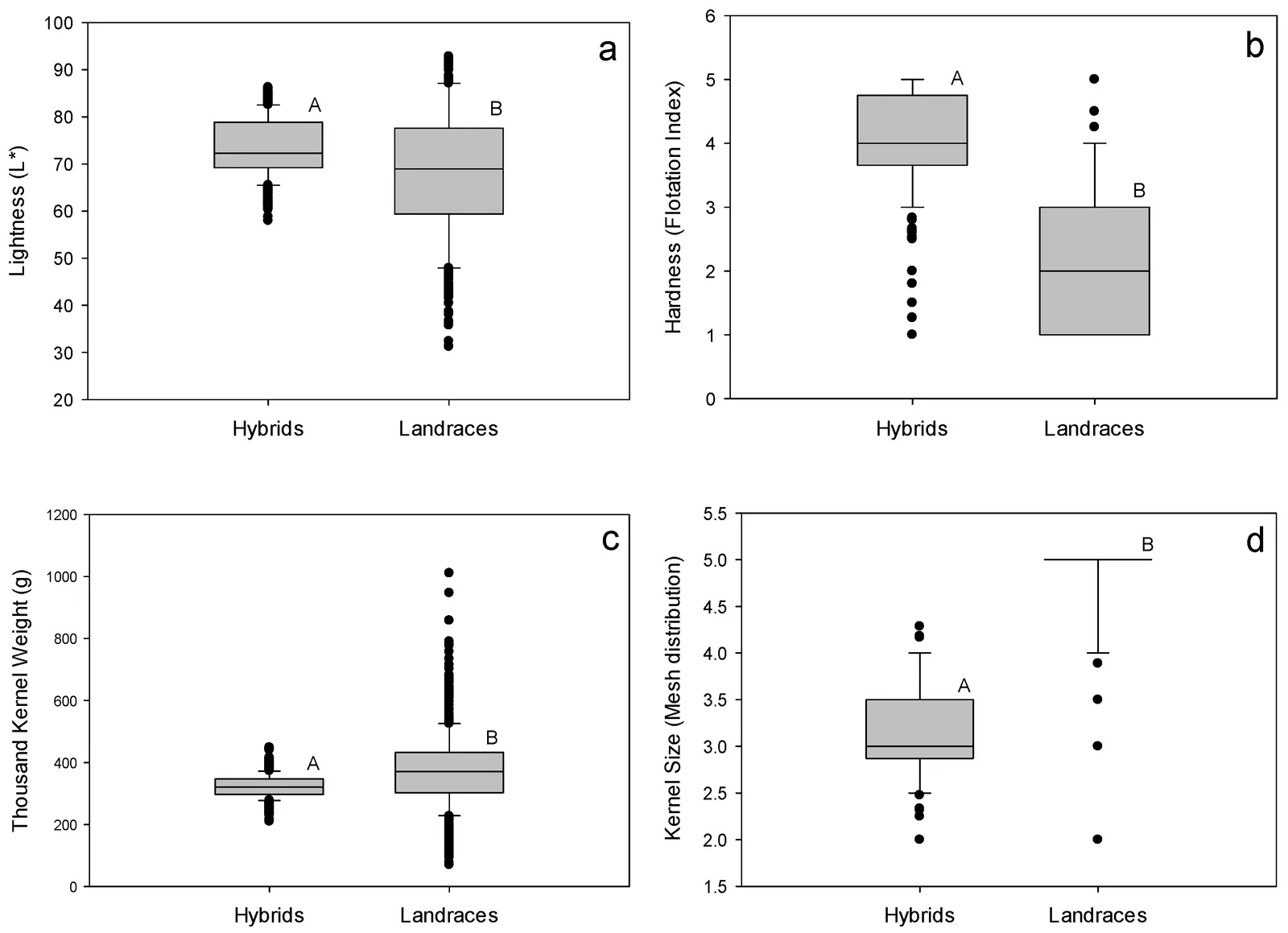
Boxplot of physical traits in hybrids and landraces: (**a**) lightness (L*), (**b**) hardness (flotation index), (**c**) thousand kernel weight (g), and (**d**) kernel size (mesh distribution). Different uppercase letters indicate statistically significant differences among groups according to ANOVA (*p* < 0.001).Collectively, these results illustrate two contrasting trajectories. Hybrids have been selected for uniformity and industrial processing characteristics, whereas landraces retain a broader spectrum of kernel phenotypes shaped by farmer selection, environmental adaptation, and cultural preferences. This diversity extends beyond morphological variation and provides opportunities to identify materials with specific nutritional and functional attributes for different food applications.

The diversity observed among landraces was reflected in their wider variation in thousand kernel weight (TKW) compared to hybrids (Figure 3). On average, landraces exhibited larger kernels, whereas hybrids showed greater uniformity, consistent with breeding strategies focused on standardized grain characteristics.

Kernel hardness was negatively correlated with kernel size (R = −0.466), a pattern also evident in Figure 3b,d, where hybrids combined higher hardness with smaller kernels, while landraces displayed the opposite trend. This contrast reflects the distinct breeding objectives and genetic backgrounds of the two groups. In contrast, a strong positive correlation was observed between kernel hardness and hectoliter weight (HW) (R = 0.694), consistent with previous reports [25].

### 3.2. Macronutrients

**Figure 4:**
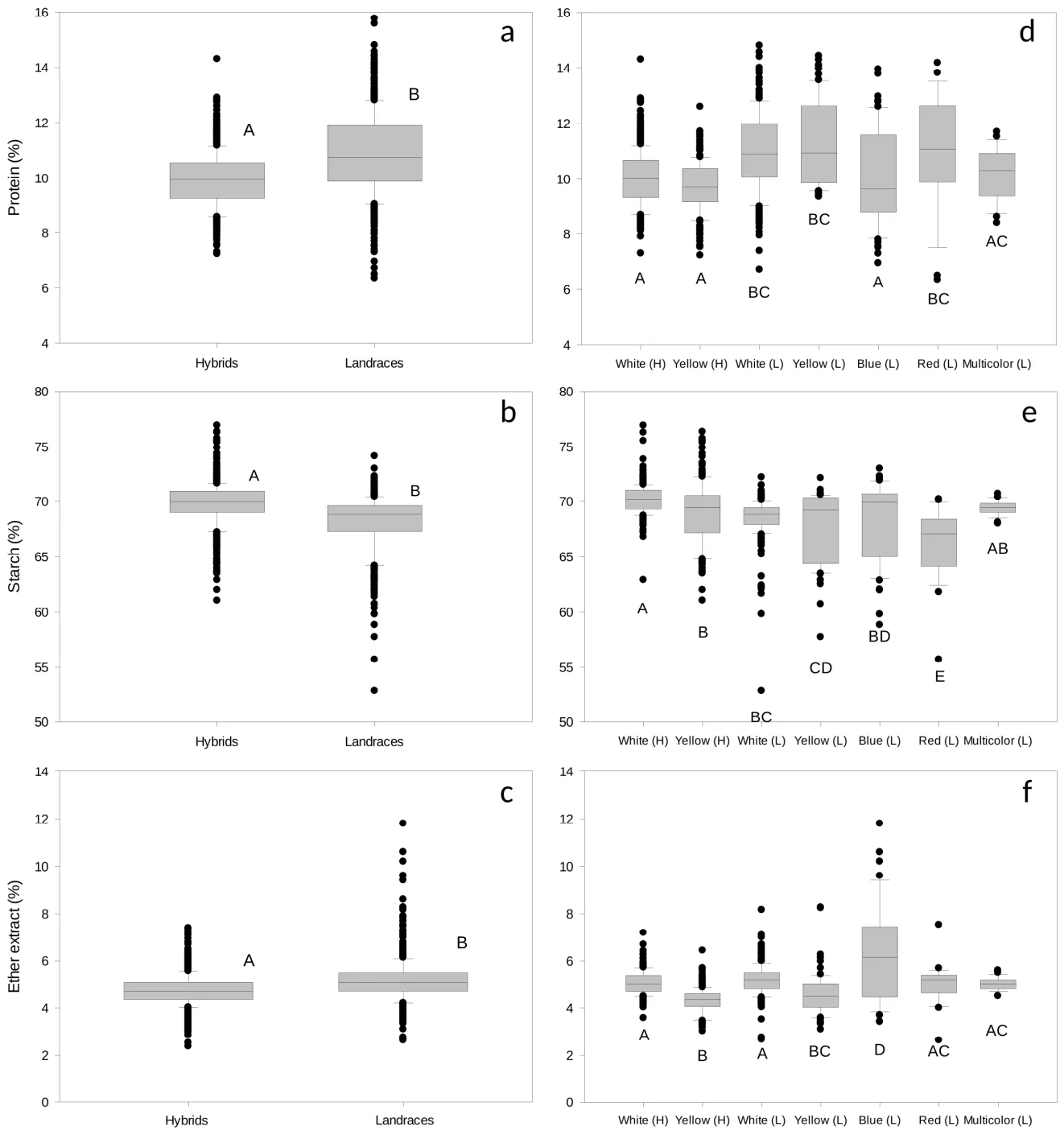
Boxplot of proximal composition in hybrids and landraces: (**a**) protein (%), (**b**) starch (%), and (**c**) ether extract (%) compared by main group; and (**d**) protein (%), (**e**) starch (%), and (**f**) ether extract (%) further detailed by kernel color and type (H = Hybrids, L = Landraces). Different uppercase letters indicate statistically significant differences among groups according to ANOVA (*p* < 0.001). Taken together, these findings indicate that the greater compositional variability observed in landraces represents an important source of genetic diversity for improving grain nutritional quality. Rather than replacing elite hybrids, landraces should be viewed as complementary genetic resources capable of broadening breeding objectives beyond yield to include nutritional value, processing quality, and adaptation to local food systems.

Comparative analysis of protein, starch, and oil content revealed significantly greater variability in macronutrient composition among landraces than among hybrids (Figure 4a–c). This increased diversity, particularly for protein and starch, reflects the broader genetic background of landraces and, to some extent, the agronomic practices commonly associated with traditional and smallholder farming systems, such as crop rotation and diversified cropping, which can influence nitrogen and carbon allocation during kernel development [4,9,12].

A significant negative correlation between protein and starch concentrations was observed (R = −0.518), consistent with previous findings in maize and other cereals. This inverse relationship suggests competition for assimilates during grain filling, whereby increases in protein accumulation are associated with reductions in starch content [41,42].

Sample origin should be considered when interpreting these results, as approximately 27.25% of samples were collected from farmers’ fields, while 72.75% originated from experimental stations where gene bank accessions had been regenerated. Variability in macronutrient content was also influenced by grain color (Figure 4d–f). Among landraces, white, yellow, and red kernels exhibited higher protein levels than blue and mixed-color kernels, which showed values closer to those observed in hybrids. In contrast, protein content in hybrids was not significantly affected by grain color, although these trends may be partly influenced by sample size and origin.

Starch content showed greater diversity in landraces than in hybrids (Figure 4b), likely associated with the broader variation in floury and vitreous endosperm proportions observed in landraces (Figure 2e,f). Starch content was negatively correlated with floury endosperm (R = −0.484) and positively correlated with vitreous endosperm (R = 0.597). In vitreous endosperm, starch granules are smaller and more densely packed within a protein-rich matrix, whereas floury endosperm is characterized by larger, loosely packed granules and higher porosity, resulting in lower starch density and softer kernel texture [41,43].

Differences in oil content were also observed between maize types. Among hybrids, white kernels exhibited higher oil content than yellow kernels, despite the latter being preferentially used in animal feed due to their higher caloric contribution [44,45]. Oil content was positively correlated with germ proportion (R = 0.312), as the germ contains approximately 50% oil and accounts for about 85% of total kernel oil and negatively correlated with endosperm proportion (R = −0.355), in agreement with the structural relationships described in Section 3.1.

Overall, landraces exhibited higher oil content than hybrids, with limited variation across grain colors, except for blue maize, which showed significantly higher oil levels. Larger kernel size and higher germ proportion in landraces likely contribute to this pattern. These results, together with reported differences in fatty acid profiles [46,47], underscore the importance of detailed germplasm characterization to fully capture the nutritional potential of maize diversity.

### 3.3. Carotenoids

**Figure 5:**
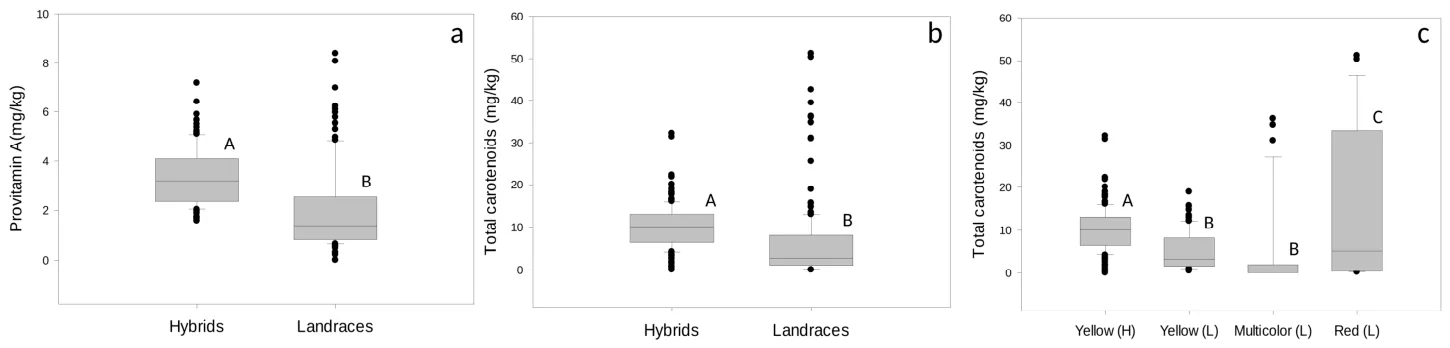
Provitamin A (**a**) and total carotenoid (**b**) boxplot in maize landraces are compared with hybrids and landraces, and (**c**) total carotenoids (mg/kg) stratified by grain color and type (H = Hybrids, L = Landraces). Different uppercase letters indicate statistically significant differences among groups according to ANOVA (*p* < 0.001).

Carotenoid content was evaluated in yellow maize samples, comparing hybrids and landraces (Figure 5). Hybrids exhibited significantly higher total carotenoid and pro-vitamin A (ProVA) concentrations, largely reflecting the inclusion of biofortified materials developed to enhance micronutrient density in maize kernels [8]. Nevertheless, landraces outperformed conventional (non-biofortified) yellow hybrids in carotenoid content, indicating that native germplasm represents an important reservoir of naturally occurring carotenoids that may contribute to future breeding efforts aimed at enhancing nutritional quality. Although average carotenoid levels were lower in landraces when all hybrids were considered together (Figure 5a), the wide range observed highlights their potential as complementary sources of these bioactive compounds.

Carotenoids are predominantly localized in the endosperm, accounting for approximately 95–97% of total kernel carotenoids, with more than 70% concentrated in the vitreous (hard) endosperm [37,41]. This distribution explains the positive correlations observed between kernel hardness and total carotenoids (R = 0.432), as well as specific compounds such as 13-cis-β-carotene (R = 0.347) and β-cryptoxanthin (R = 0.340). Harder kernels, characterized by a higher proportion of vitreous endosperm, tend to accumulate greater carotenoid concentrations, consistent with previous reports [37,48].

Significant associations were also detected between carotenoids and major storage components. Starch content was positively correlated with several carotenoid fractions, including 9-cis-β-carotene (R = 0.404), 13-cis-β-carotene (R = 0.453), β-carotene (R = 0.346), and ProVA (R = 0.370), whereas protein content showed strong negative correlations with total and provitamin A carotenoids (R = −0.446 to −0.589). These relationships likely reflect interactions within the starch–protein matrix that determine kernel hardness and endosperm compactness [37,42].

Oil content was negatively correlated with total carotenoids (R = −0.455), a pattern consistent with the anatomical distribution of lipids and pigments in maize kernels. As most crude fat is concentrated in the germ, lower oil content may indicate a smaller germ and a proportionally larger carotenoid-rich endosperm. This interpretation aligns with the negative association between germ and endosperm proportions described in Section 3.1 and with previous studies linking reduced germ size to higher relative carotenoid concentration in the endosperm [49].

Notably, some multicolor and red landraces exhibited relatively high total carotenoid levels, underscoring their value as underexplored genetic resources. Incorporating such landraces into breeding programs could enhance carotenoid diversity while supporting adaptation, adoption, and stability of improved varieties, particularly in regions where maize diversity and cultural relevance remain high.

The nutritional contribution of carotenoids is not determined solely by their concentration but also by their stability and accessibility during processing. Kernel structure, lipid content, and the starch–protein matrix influence carotenoid retention and release, while processing methods such as milling, cooking, and fermentation may differentially affect carotenoid degradation and bioaccessibility. Thus, both genetic diversity and grain structural traits play a key role in shaping the effective contribution of carotenoids in maize-based diets.

These results further demonstrate how targeted breeding and natural diversity contribute differently to nutritional improvement. Biofortification programs have successfully increased provitamin A concentrations in elite germplasm, whereas landraces provide a complementary source of carotenoid diversity that may contribute novel alleles, broader adaptation, and improved acceptance in regions where traditional varieties are still predominant.

### 3.4. Anthocyanins

**Figure 6:**
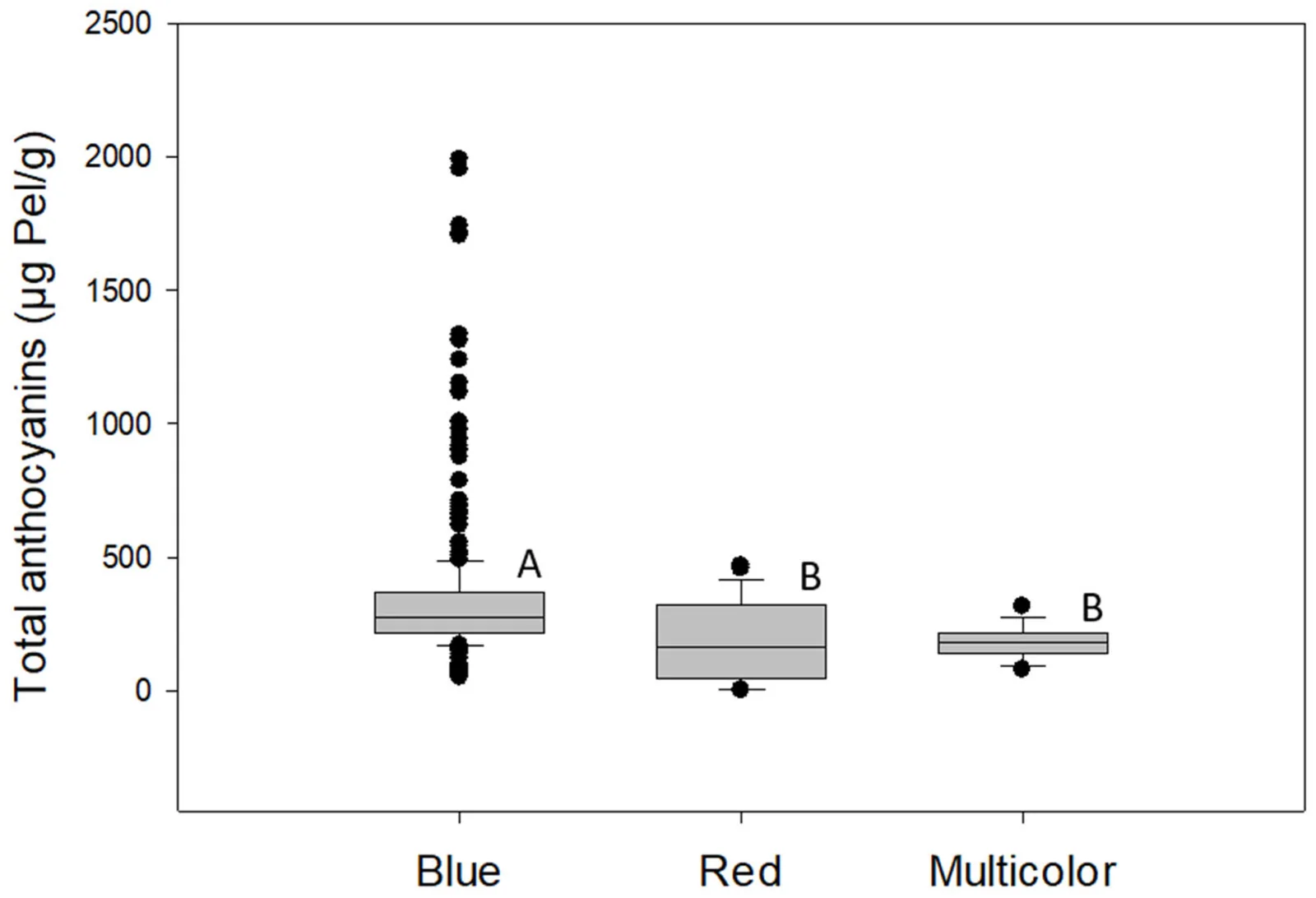
Anthocyanins boxplot in maize landraces. Different uppercase letters indicate statistically significant differences among groups according to ANOVA (*p* < 0.001).

All blue, red, and multicolored grains analyzed in this study originated exclusively from landraces. As shown in Figure 6, total anthocyanin content exhibited substantial variation, with blue maize displaying consistently higher concentrations than red and multicolored varieties. This pattern agrees with previous studies reporting elevated anthocyanin levels in blue maize, particularly cyanidin- and pelargonidin-based compounds, as well as a broader diversity of anthocyanin profiles compared with other pigmented maize types [6,23].

The observed variation in anthocyanin content among landraces reflects both genetic and structural factors. Anthocyanins are primarily localized in the pericarp and aleurone layers, and their concentration can be influenced by kernel size through a dilution effect when expressed on a flour basis. In general, higher anthocyanin concentrations are associated with the pericarp of purple maize and the aleurone layer of blue maize [6,50]. However, in the present study, the ability to directly assess relationships between anthocyanin content, kernel size, and kernel structural components was limited by the reduced overlap among these measurements.

Blue maize showed both greater variability and higher average anthocyanin content than red and multicolored maize (Figure 6). It should be noted that the number of blue maize samples was substantially larger than that of red and multicolored samples (886, 48, and 33, respectively), which may partially influence the magnitude of the observed differences. Nevertheless, these results are consistent with previous reports indicating that red maize generally contains lower anthocyanin concentrations than purple or magenta-colored grains [50].

From an application perspective, this information is relevant for identifying pigmented maize types with higher nutraceutical potential for food and non-food uses, including functional foods, natural colorants, and specialty ingredients.

Significant correlations were detected between total anthocyanins and major storage components, including protein (R = −0.539), starch (R = 0.494), and oil content (R = 0.556). While the trends observed for protein and starch were similar to those reported for carotenoids, the positive association with oil content followed an opposite pattern. The underlying mechanisms driving these relationships remain unclear and may involve kernel size effects, anatomical localization of anthocyanins, or interactions among storage tissues. Due to the lack of detailed data on anthocyanin distribution within kernel fractions, these hypotheses could not be directly evaluated in this study.

Evidence from other crops suggests that the relationship between anthocyanins and oil content may vary by tissue and species. For example, negative correlations between oil and anthocyanins have been reported in peanuts [51], whereas recent findings in maize indicate that pigmented germ tissue may contribute substantially—up to 50%—to total kernel anthocyanins [52]. These contrasting observations highlight the need for further research integrating biochemical, anatomical, and genetic analyses to better understand anthocyanin accumulation in maize kernels.

The nutraceutical potential of anthocyanin-rich maize depends not only on total anthocyanin content but also on their localization within kernel tissues and their stability during processing. Anthocyanins concentrated in the pericarp, aleurone, and, in some cases, the germ may respond differently to processing, influencing their retention and functional properties in final products. Consequently, expanding analyses beyond whole-kernel flour to specific kernel fractions with higher pigment concentration represents an important opportunity to better exploit pigmented maize, particularly in the development of natural colorants and value-added food and non-food applications. The substantial variation observed among pigmented landraces also suggests opportunities to diversify the utilization of maize beyond staple foods. Anthocyanin-rich materials may support the development of functional foods, natural pigments, and specialty products with added nutritional and economic value.

### 3.5. Iron and Zinc

Zinc (Zn) is an essential micronutrient, functioning as a structural component of numerous proteins, including transcription factors and metalloenzymes, and playing a critical role in both plant and human nutrition. Although kernel Zn concentration is influenced by genetic and environmental factors such as soil conditions, substantial genetic variation for Zn accumulation has been documented in maize and other cereals. Consequently, several breeding programs have focused on developing high-Zn maize hybrids with improved agronomic performance and adaptation [8].

**Figure 7:**
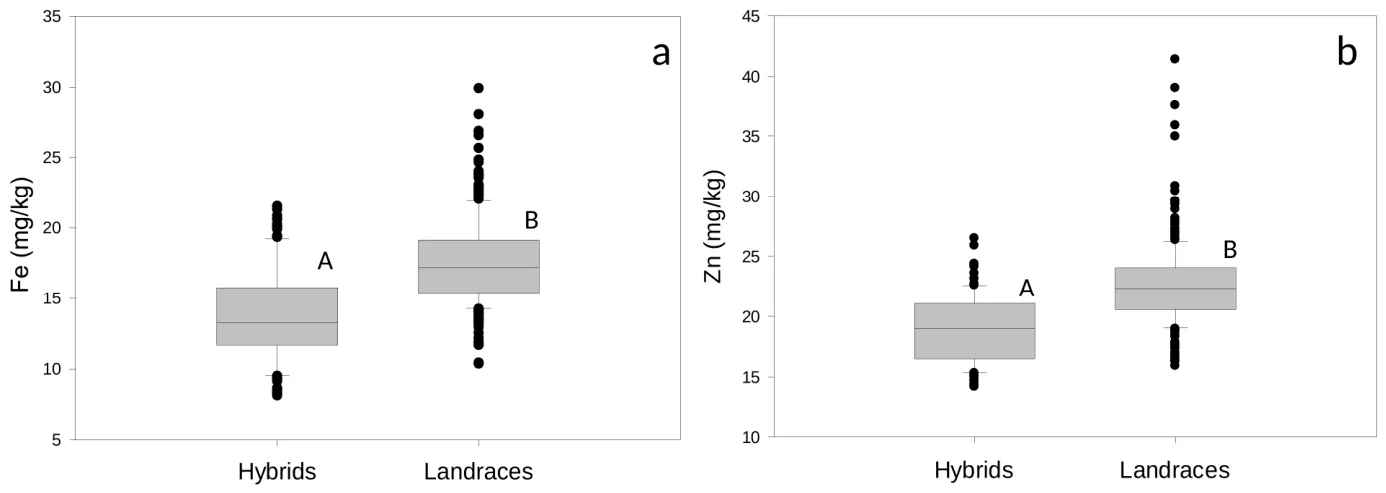
Boxplot comparison of mineral content in maize: (**a**) iron (Fe, mg/kg) and (**b**) zinc (Zn, mg/kg) between hybrids and landraces. Different uppercase letters indicate statistically significant differences among groups according to ANOVA (*p* < 0.001).

In the present study, landraces exhibited higher average Zn content (22.69 ± 3.57 mg kg^−1^) than hybrids (18.98 ± 2.96 mg kg^−1^), highlighting the value of landrace germplasm as a source of alleles for micronutrient enhancement (Figure 7). Across all samples, Zn content averaged 21.60 ± 3.78 mg kg^−1^, with a wide range (14.17–41.38 mg kg^−1^) consistent with values reported in diverse maize germplasm worldwide [3,8,33,53,54].

A similar pattern was observed for iron (Fe), with landraces showing higher average concentrations (29.95 ± 3.14 mg kg^−1^) than hybrids (21.55 ± 3.29 mg kg^−1^) (Figure 7). The range of Fe values observed in this study (14.17–41.38 mg kg^−1^) aligns with previous reports for maize [3,33,53,54]. Despite this variability, maize has not been a primary target for Fe biofortification, as kernel Fe concentrations are generally insufficient to achieve a major nutritional impact and are further constrained by the presence of bioavailability inhibitors in processed maize products [33].

Notably, increasing Zn concentration has been shown to concurrently enhance Fe content, a relationship reported in several studies and confirmed in the present work by a significant positive correlation between Zn and Fe (R = 0.483). This association suggests shared or closely linked physiological pathways governing Zn and Fe accumulation in maize kernels, offering opportunities for simultaneous improvement of both micronutrients through breeding [54,55].

Both Zn and Fe contents were positively correlated with kernel size (R = 0.446 and 0.427, respectively), consistent with reports indicating higher micronutrient concentrations in larger kernels [40,56]. This trend may be partly explained by the greater proportion of germ in larger grains, as the germ represents an important reservoir for Zn. However, micronutrient distribution within the kernel is shaped by complex interactions between genetic background and environmental conditions, underscoring the need to consider both factors when evaluating Zn and Fe accumulation [33,55,57].

A significant positive correlation between protein content and Zn concentration (R = 0.347) was also observed, in agreement with previous studies. This relationship indicates that Zn biofortification may influence other nutritional and antinutritional traits, reinforcing the importance of integrated nutritional profiling when developing biofortified maize varieties [55].

Overall, these results highlight the complementarity between targeted biofortification strategies—particularly effective for Zn—and the conservation and characterization of landrace diversity, which remains a critical reservoir of micronutrient variation. Leveraging both approaches may enhance the nutritional quality, resilience, and adoption potential of future maize cultivars. These findings further illustrate the value of germplasm collections as reservoirs of nutritionally valuable variation. Accessions already characterized for micronutrient traits can be incorporated into breeding and research programs, while the large number of uncharacterized materials conserved in gene banks may harbor additional alleles and trait combinations of relevance for future biofortification efforts.

Beyond total concentration, the nutritional relevance of Zn and Fe is strongly influenced by their bioavailability, which depends on kernel composition and processing practices. Factors such as phytate content, protein matrix organization, germ proportion, and endosperm structure can modulate mineral absorption, while different processing methods may alter mineral retention and interactions with antinutritional compounds. Therefore, evaluating Zn and Fe in maize requires an integrated consideration of genetic background, kernel structure, and processing conditions to better understand their nutritional contribution in maize-based foods.

Together, these observations reinforce the complementary roles of modern breeding and germplasm conservation. While biofortification programs effectively enhance micronutrient density in elite germplasm, landraces continue to represent a valuable reservoir of natural variation that may accelerate future breeding efforts aimed at improving nutritional quality while maintaining adaptation to diverse production environments.

### 3.6. Selection of Maize Samples with High Nutritional Values

To complement the variability analyses, maize samples with the highest values for iron (Fe), zinc (Zn), total carotenoids, and anthocyanins were identified from the full dataset (Table 3). This selection does not represent a separate analytical approach, but rather highlights samples located at the upper end of the observed nutritional ranges.

Most of the samples with the highest Fe and Zn concentrations corresponded to landraces, particularly those from traditional farming systems in Central and Southern Mexico, underscoring their relevance as sources of micronutrient-rich germplasm. In contrast, samples with higher provitamin A carotenoid content were mainly hybrids, reflecting differences in selection history and breeding objectives. Pigmented landraces, especially blue and red maize, predominated among samples with higher anthocyanin content.

Overall, this selection illustrates the contrasting and complementary nutritional strengths observed between landraces and hybrids and supports the study’s objective of documenting maize compositional diversity relevant to food quality, breeding, and utilization.

Although the materials analyzed originated from different years, locations, and production systems, all samples were evaluated under standardized laboratory protocols at CIMMYT. Consequently, the observed variation reflects both genetic diversity and environmental influences. Rather than attempting to partition these effects, the objective of this study was to document the breadth of compositional diversity available within maize germplasm and to identify nutritional patterns that may guide future breeding, food innovation, and targeted experimentation under controlled environments.

## 4. Conclusions

This study highlights the importance of maize genetic diversity for shaping nutritional composition, kernel structure, and potential food applications. By integrating physical traits, macronutrients, bioactive compounds, and micronutrients, our results demonstrate that landraces and hybrids offer complementary nutritional profiles rather than interchangeable solutions. While landraces tend to exhibit greater variability and, on average, higher concentrations of minerals, lipids, and phytochemicals, hybrids generally provide more uniform starch and carotenoid profiles.

Although previous studies have emphasized the superior diversity of landraces, our findings underscore the need for detailed, case-by-case characterization to capture meaningful nutritional variation and to ensure agronomic relevance. The associations observed between kernel structures and nutrient density further suggest that traits such as germ proportion, pericarp thickness, and endosperm type could serve as practical indicators for nutritional quality and may be leveraged in selection strategies.

Importantly, nutritional value cannot be fully assessed based on concentration alone. Kernel anatomy, genetic background, and processing conditions jointly determine nutrient retention, bioaccessibility, and functionality in maize-based foods. In this context, pigmented landraces rich in phytochemicals represent valuable resources for developing differentiated food products, natural colorants, and niche markets, particularly where cultural preferences and traditional uses influence adoption.

Overall, leveraging maize diversity through integrated characterization, while accounting for processing and cultural relevance, can support more resilient, nutritionally diverse, and context-specific food systems. The substantial genetic diversity conserved in germplasm collections, such as the CIMMYT Genebank, provides unique opportunities to identify and deploy nutritionally valuable materials for breeding, food innovation, and research. This resource can be leveraged both through expanded characterization of underexplored accessions and through the direct utilization of already characterized germplasm with desirable nutritional and functional traits. Beyond conservation, germplasm banks should increasingly be viewed as dynamic platforms for identifying, characterizing, and deploying nutritional diversity to address future food, health, and sustainability challenges.

Future studies conducted under common environments will be necessary to disentangle genetic and environmental effects on grain quality traits. And additional research should combine compositional analyses with sensory evaluation and participatory approaches to better align breeding and utilization strategies with real-world demand. Expanding characterization efforts to include additional bioactive and functional compounds, as well as grain functionality and constituents traditionally classified as antinutritional factors, may further enhance our understanding of the nutritional and functional diversity present in maize germplasm.

## Figures and Tables

**Table 1:** Scale to classify grain physical traits.

Scale	Color	Size	Hardness
1	White	Very small	Very soft
2	Yellow	Small	Soft
3	Blue	Intermediate	Intermediate
4	Red	Large	Hard
5	Mixed	Very large	Very hard

**Table 2:** Variability of physical, chemical, and nutritional compounds in hybrids and landraces.

	Hybrids				Landraces			
Trait	Number of Samples	Maximum	Minimum	Average	Number of Samples	Maximum	Minimum	Average
Color classification	554	2	1	1	773	5	1	2
L value	485	86.24	57.93	73.54	461	92.785	31.22	68.09
a value	485	18.11	0.48	6.12	459	26.035	−0.91	4.94
b value	485	55.03	17.26	31.06	459	47.165	−1.84	24.59
TKW, g	490	448.8	209.8	322.5	722	1011.1	70.5	374.7
Hardness classification	490	5	1	4	729	5	1	2
Hectoliter weight, kg hL^−1^	111	83.02	72.50	78.37	0	NA	NA	NA
Tip cap, %	314	4.63	0.43	1.75	60	6.79	1.18	3.40
Pericarp, %	314	7.60	3.54	5.22	62	13.41	2.89	4.80
Germ, %	314	17.56	7.39	11.42	60	18.12	6.25	10.81
Floury endosperm, %	42	84.01	14.29	36.53	53	83.95	5.27	54.93
The vitreous endosperm, %	38	67.27	26.75	51.86	53	78.30	0	25.82
Endosperm, %	314	85.27	75.99	81.60	62	87.08	70.39	81.04
Kernel size classification	274	4	2	3	226	5	2	5
Protein, %	519	14.30	7.23	9.92	510	15.76	6.36	10.88
Trp, %	0	NA	NA	NA	212	0.081	0.001	0.055
Lys, %	0	NA	NA	NA	200	0.459	0.126	0.335
Starch, %	506	76.92	61.05	69.76	486	74.12	52.85	68.06
Ether extract, %	519	7.20	3.02	4.77	472	11.80	2.63	5.20
Total anthocyanins, µg Pel g^−1^	0	NA	NA	NA	438	1990.0	0.4	289.7
Lutein, µg g^−1^	115	5.29	0.24	1.70	122	10.67	0	1.25
Zeaxanthin, µg g^−1^	115	17.58	0.74	4.81	122	27.72	0.11	2.99
β-Cryptoxanthin, µg g^−1^	115	5.40	0.30	2.05	122	9.53	0	1.30
13-cis-β-carotene, µg g^−1^	115	0.86	0.25	0.48	122	0.81	0	0.28
β-Carotene, µg g^−1^	115	2.66	0.47	1.21	122	2.26	0	0.59
9-cis-β-carotene, µg g^−1^	115	1.26	0.29	0.62	122	0.93	0	0.31
Provitamin A, µg g^−1^	115	7.18	1.57	3.33	122	8.40	0	1.84
Total carotenoids, µg g^−1^	182	32.24	0	10.03	339	51.23	0	3.23
Fe, mg kg^−1^	93	21.55	8.09	13.91	225	29.95	10.38	17.63
Zn, mg kg^−1^	93	26.49	14.18	18.98	225	41.39	15.89	22.70

Abbreviation: NA, not available. Classification scale is presented in Table 1.

**Table 3:** Selection of landraces and hybrids with high content of key nutritional traits.

Type	Sample Code *	Key Nutritional Trait	Value	Unit
Hybrid	CHLHW12013	Protein	15.3	%
Hybrid	CHLHW14003	Protein	14.4	%
Hybrid	CLTHY11002	Starch	76.81	%
Hybrid	CHLHY09004	Starch	75.83	%
Hybrid	ODIN-B	Ether extract	7.4	%
Hybrid	H388-O	Ether extract	7.35	%
Hybrid	CLTHY14002	Total carotenoids	31.64	µg g^−1^
Hybrid	CHLHW12013	Fe	25.3	mg kg^−1^
Hybrid	RS1021	Fe	25	mg kg^−1^
Hybrid	CLTHW15001	Zn	30.03	mg kg^−1^
Hybrid	CLTHW15007	Zn	29.8	mg kg^−1^
Landrace	HIDA 131	Protein	15.76	%
Landrace	JALI 300	Protein	15.57	%
Landrace	BM San Juan	Starch	73	%
Landrace	Nativo 3 (Santa Cruz)	Starch	72.56	%
Landrace	PBS-002	Ether extract	11.8	%
Landrace	PBS-011	Ether extract	10.6	%
Landrace	URUG,228A-#	Total carotenoids	51.23	µg g^−1^
Landrace	URUG,177A-B	Total carotenoids	42.58	µg g^−1^
Landrace	CON-101	Fe	53.31	mg kg^−1^
Landrace	PBS-046	Fe	52.17	mg kg^−1^
Landrace	CR. PISCORUNTO II	Zn	45.22	mg kg^−1^
Landrace	PBS-013	Zn	41.41	mg kg^−1^

* Germplasm available from CIMMYT GenBank (https://maizecatalog.cimmyt.org/). Accessed on 1 March 2026.

## Data Availability

The original contributions presented in this study are included in the article/Supplementary Materials. Further inquiries can be directed to the corresponding author.

## Supplementary Materials

The following supporting information can be downloaded at: https://www.mdpi.com/article/10.3390/foods15162933/s1, Table S1. The correlations between the physical and nutritional characteristics of the samples analyzed were assessed. However, due to the large volume of data, only those statistically significant correlations, as determined by the R coefficient, *p*-value, and sample size, are reported in the table: Highlighted correlations between physical and nutritional traits of maize.

## Abbreviations

The following abbreviations are used in this manuscript: TKW, thousand kernel weight; HW, hectoliter weight; Lys, lysine; trp, tryptophan; Fe, iron; Zn, zinc; L, luminosity; Lut, lutein; Zea, zeaxanthin; βcry, β-cryptoxanthin; βcar, β-carotene; 9cis, 9-cis-β-carotene; 13cis, 13-cis-β-carotene; proVA, provitamin A carotenoids; UPLC, ultra-performance liquid chromatography; NIRS, near-infrared reflectance spectroscopy; ICP-OES, inducted coupled plasma optical emission spectroscopy; XRF, X-ray fluorescence; PAM, Peruvian Andean maize.

## References

[1] González-Amaro R.M., de Dios Figueroa-Cárdenas J., Perales H., Santiago-Ramos D. (2015). Maize races on the functional and nutritional quality of tejate: A maize-cacao beverage. LWT-Food Sci. Technol..

[2] Guzzon F., Rios L.W.A., Cepeda G.M.C., Polo M.C., Cabrera A.C., Figueroa J.M., Hoyos A.E.M., Calvo T.W.J., Molnar T.L., León L.A.N. (2021). Conservation and use of Latin American maize diversity: Pillar of nutrition security and cultural heritage of humanity. Agronomy.

[3] Palacios-Rojas N., McCulley L., Kaeppler M., Titcomb T.J., Gunaratna N.S., Lopez-Ridaura S., Tanumihardjo S.A. (2020). Mining maize diversity and improving its nutritional aspects within agro-food systems. Compr. Rev. Food Sci. Food Saf..

[4] Cabrera-Soto L., Pixley K.V., Rosales-Nolasco A., Galicia-Flores L.A., Palacios-Rojas N. (2018). Carotenoid and Tocochromanol Profiles during Kernel Development Make Consumption of Biofortified “Fresh” Maize an Option to Improve Micronutrient Nutrition. J. Agric. Food Chem..

[5] Jambrović A., Šimić D., Ledenčan T., Zdunić Z., Brkić I. (2008). Genetic diversity among maize (*Zea mays*, L.) inbred lines in Eastern Croatia. Period. Biol..

[6] Paulsmeyer M.N., Juvik J.A. (2023). Increasing aleurone layer number and pericarp yield for elevated nutrient content in maize. G3 Genes Genomes Genet..

[7] Cairns J.E., Prasanna B.M. (2018). Developing and deploying climate-resilient maize varieties in the developing world. Curr. Opin. Plant Biol..

[8] Prasanna B.M., Palacios-Rojas N., Hossain F., Muthusamy V., Menkir A., Dhliwayo T., Ndhlela T., San Vicente F., Nair S.K., Vivek B.S. (2020). Molecular Breeding for Nutritionally Enriched Maize: Status and Prospects. Front. Genet..

[9] Willcox M.C., Castillo-Gonzalez F., Aragón-Cuevas F., Castro-Garcia F.H. (2019). Native maize in Mexico. Farmers and Plant Breeding.

[10] Ekpa O., Palacios-Rojas N., Kruseman G., Fogliano V., Linnemann A.R. (2018). Sub-Saharan African maize-based foods: Technological perspectives to increase the food and nutrition security impacts of maize breeding programmes. Glob. Food Secur..

[11] Álvarez-Iglesias L., Malvar R.A., Garzón R., Rosell C.M., Revilla P. (2021). Nutritional value of whole maize kernels from diverse endosperm types and effects on rheological quality. Agronomy.

[12] Ibba M.I., Palacios-Rojas N., Rosales-Nolasco A. (2023). Screening and use of nutritional and health-related benefits of the main crops. Developing Sustainable and Health-Promoting Cereals and Pseudocereals: Conventional and Molecular Breeding.

[13] Langyan S., Bhardwaj R., Kumari J., Jacob S.R., Bisht I.S., Pandravada S.R., Singh A., Singh P.B., Dar Z.A., Kumar A. (2022). Nutritional Diversity in Native Germplasm of Maize Collected From Three Different Fragile Ecosystems of India. Front. Nutr..

[14] Nelimor C., Badu-Apraku B., Garcia-Oliveira A.L., Tetteh A., Paterne A., N’guetta A.S.P., Gedil M. (2020). Genomic analysis of selected maize landraces from Sahel and coastal west Africa reveals their variability and potential for genetic enhancement. Genes.

[15] Preciado-Ortiz R.E., Ochoa-Centeno N.J., Vázquez-Carrillo M.G., Santiago-Ramos D., Terrón-Ibarra A.D. (2022). Grain yield, physical and pasting properties, and anthocyanins of non-conventional pigmented corn hybrids for pozole end-use adapted to subtropical regions. Appl. Food Res..

[16] Vázquez-Carrillo M.G., Santiago-Ramos D., Domínguez-Rendón E., Audelo-Benites M.A. (2017). Effects of two different Pozole preparation processes on quality variables and pasting properties of processed maize grain. J. Food Qual..

[17] Acosta-Estrada B.A., Serna-Saldívar S.O., Chuck-Hernández C. (2023). Nutritional assessment of nixtamalized maize tortillas produced from dry masa flour, landraces, and high-yield hybrids and varieties. Front. Nutr..

[18] Domínguez-Hernández E., Gaytán Martínez M., Gutiérrez-Uribe J.A., Domínguez-Hernández M.E. (2022). The nutraceutical value of maize (*Zea mays* L.) landraces and the determinants of its variability: A review. J. Cereal Sci..

[19] Dorantes-Campuzano M.F., Cabrera-Ramírez A.H., Rodríguez-García M.E., Palacios-Rojas N., Preciado-Ortíz R.E., Luzardo-Ocampo I., Gaytán Martínez M. (2022). Effect of maize processing on amylose-lipid complex in pozole, a traditional Mexican dish. Appl. Food Res..

[20] Gálvez-Ranilla L., Rios-Gonzales B.A., Ramírez-Pinto M.F., Fuentealba C., Pedreschi R., Shetty K. (2021). Primary and phenolic metabolites analyses, in vitro health-relevant bioactivity, and physical characteristics of purple corn (*Zea mays* L.) grown at two Andean geographical locations. Metabolites.

[21] Gaytán-Martínez M., Figueroa-Cárdenas J.D., Reyes-Vega M.L., Morales-Sánchez E., Rincón-Sánchez F. (2013). Maize landraces selection for industrial end-use based on their added value | Selección de maíces criollos para su aplicación en la industria con base en su valor agregado. Rev. Fitotec. Mex..

[22] Mora-Rochin S., Gutiérrez-Uribe J.A., Serna-Saldivar S.O., Sánchez-Peña P., Reyes-Moreno C., Milán-Carrillo J. (2010). Phenolic content and antioxidant activity of tortillas produced from pigmented maize processed by conventional nixtamalization or extrusion cooking. J. Cereal Sci..

[23] Peniche-Pavía H.A., Tiessen A. (2020). Anthocyanin Profiling of Maize Grains Using DIESI-MSQD Reveals That Cyanidin-Based Derivatives Predominate in Purple Corn, whereas Pelargonidin-Based Molecules Occur in Red-Pink Varieties from Mexico. J. Agric. Food Chem..

[24] Peralta-Veran L., Espinosa-Leal C., Escalante-Aburto A., Preciado-Ortiz R.E., Puente-Garza C.A., Serna-Saldivar S.O., García-Lara S. (2022). Effects of pozole broth production on phenolic acids and antioxidant activity of specialty maize landraces. J. Cereal Sci..

[25] Vázquez-Carrillo G., García-Lara S., Salinas-Moreno Y., Bergvinson D.J., Palacios-Rojas N. (2011). Grain and tortilla quality in landraces and improved maize grown in the highlands of Mexico. Plant Foods Hum. Nutr..

[26] Camelo-Méndez G.A., Agama-Acevedo E., Tovar J., Bello-Pérez L.A. (2017). Functional study of raw and cooked blue maize flour: Starch digestibility, total phenolic content and antioxidant activity. J. Cereal Sci..

[27] Odjo S., Palacios-Rojas N., Burgueño J., Corrado M., Ortner T., Verhulst N. (2022). Hermetic storage technologies preserve maize seed quality and minimize grain quality loss in smallholder farming systems in Mexico. J. Stored Prod. Res..

[28] Taleon V., Mugode L., Cabrera-Soto L., Palacios-Rojas N. (2017). Carotenoid retention in biofortified maize using different post-harvest storage and packaging methods. Food Chem..

[29] Palacios-Rojas N. (2018). Calidad Nutricional e Industrial de Maíz. Protocolos.

[30] Hernández-Quintero J.D., Rosales-Nolasco A., Molina-Macedo A., Miranda-Piliado A., Willcox M., Hernández-Casillas J.M., Palacios-Rojas N. (2017). Quantification of anthocyanins through near-infrared spectroscopy and liquid chromatography in pigmented maize. Rev. Fitotec. Mex..

[31] Molina-Macedo A., Basilio-Hernández R., Rosas-Sánchez I., Rosales-Nolasco A., Palacios-Rojas N. (2023). Assessment of Maize Grain Hardness Using Sugar Solution for Flotation Index Determination.

[32] Molina-Macedo A., Galicia-Flores L.A., Rosales-Nolasco A., Palacios-Rojas N. (2023). Determination of total starch in maize flour using a modified Megazyme assay. Maize Total Starch Determination (Lab Protocol).

[33] Rosales-Nolasco A., Crossa J., Cuevas J., Cabrera-Soto L., Dhliwayo T., Ndhlela T., Palacios-Rojas N. (2022). Near-Infrared Spectroscopy to Predict Provitamin A Carotenoids Content in Maize. Agronomy.

[34] Rosales-Nolasco A., Molina-Macedo A., Leyva M., San Vicente F., Palacios-Rojas N. (2023). Fresh/High-Zinc Maize: A Promising Solution for Alleviating Zinc Deficiency through Significant Micronutrient Accumulation. Foods.

[35] Cabrera-Soto L., Rosales-Nolasco A., Molina-Macedo A., Vázquez-Carrillo G., Palacios-Rojas N. (2023). Analysis of Grain Characteristics of Commercial Maize Hybrids in Mexico: A Comprehensive Perspective on Grain Quality in the Last Decade.

[36] Mariani N.C.T., de Almeida Teixeira G.H., de Lima K.M.G., Morgenstern T.B., Nardini V., Júnior L.C.C. (2015). NIRS and iSPA-PLS for predicting total anthocyanin content in jaboticaba fruit. Food Chem..

[37] Saenz E., Borras L., Gerde J.A. (2021). Carotenoid profiles in maize genotypes with contrasting kernel hardness. J. Cereal Sci..

[38] Twumasi-Afriyie S., Palacios-Rojas N., Friesen D., Teklewold A., Wegary D., De Groote H., Prasanna B.M. (2016). Guidelines for the Quality Control of Quality Protein Maize (QPM) Seed and Grain.

[39] Medina-Hoyos A., Narro-León L.A., Chávez-Cabrera A. (2020). Purple corn (*Zea mays* L.) crop in the Peruvian Highlands: Adaptation and identification of high yield and anthocyanin content cultivars. Sci. Agropecu..

[40] Zhang H., Xu G. (2019). Physicochemical properties of vitreous and floury endosperm flours in maize. Food Sci. Nutr..

[41] Kljak K., Duvnjak M., Grbeša D. (2018). Contribution of zein content and starch characteristics to vitreousness of commercial maize hybrids. J. Cereal Sci..

[42] Wang S., Chao C., Cai J., Niu B., Copeland L., Wang S. (2020). Starch–lipid and starch–lipid–protein complexes: A comprehensive review. Compr. Rev. Food Sci. Food Saf..

[43] Zurak D., Kljak K., Grbeša D. (2020). The composition of floury and vitreous endosperm affects starch digestibility kinetics of the whole maize kernel. J. Cereal Sci..

[44] Del Pozo-Insfran D., Brenes C.H., Serna-Saldivar S.O., Talcott S.T. (2006). Polyphenolic and antioxidant content of white and blue corn (*Zea mays* L.) products. Food Res. Int..

[45] Libron J.A.M.A., Cardona D.E.M., Mateo J.M.C., Beltran A.K.M., Tuaño A.P.P., Laude T.P. (2021). Nutritional properties and phenolic acid profile of selected Philippine pigmented maize with high antioxidant activity. J. Food Compos. Anal..

[46] Preciado-Ortiz R.E., García-Lara S., Ortiz-Islas S., Ortega-Corona A., Serna-Saldivar S.O. (2013). Response of recurrent selection on yield, kernel oil content and fatty acid composition of subtropical maize populations. Field Crops Res..

[47] Yang X., Guo Y., Yan J., Zhang J., Song T., Rocheford T., Li J.S. (2010). Major and minor QTL and epistasis contribute to fatty acid compositions and oil concentration in high-oil maize. Theor. Appl. Genet..

[48] Saenz E., Abdala L.J., Borrás L., Gerde J.A. (2020). Maize kernel color depends on the interaction between hardness and carotenoid concentration. J. Cereal Sci..

[49] Wang H., Huang Y., Xiao Q., Huang X., Li C., Gao X., Wang Q., Xiang X., Zhu Y., Wang J. (2020). Carotenoids modulate kernel texture in maize by influencing amyloplast envelope integrity. Nat. Commun..

[50] Salinas-Moreno Y., Pérez-Alonso J.J., Vázquez-Carrillo G., Aragón-Cuevas F., Velázquez-Cardelas G.A. (2012). Antocianinas y actividad antioxidante en maíces (*Zea mays* L.) de las razas chalqueño, elotes cónicos y bolitas. Agrociencia.

[51] Kuang Q., Yu Y., Attree R., Xu B. (2017). A comparative study on anthocyanin, saponin, and oil profiles of black and red seed coat peanut (*Arachis hypogacea*) grown in China. Int. J. Food Prop..

[52] Vázquez-Carrillo M.G., Palos-Hernández A., González-Paramás A.M., Santos-Buelga C., García-Cruz L., Arellano-Vázquez J.L., Rojas-Martínez I. (2025). Blue maize with pigmented germ: Phytochemical compounds and tortilla color. Food Chem..

[53] Ortiz-Monasterio J.I., Palacios-Rojas N., Meng E., Pixley K., Trethowan R., Pena R.J. (2007). Enhancing the mineral and vitamin content of wheat and maize through plant breeding. J. Cereal Sci..

[54] Udo E., Abe A., Meseka S., Mengesha W., Menkir A. (2023). Genetic Analysis of Zinc, Iron and Provitamin A Content in Tropical Maize (*Zea mays* L.). Agronomy.

[55] Akinwale R.O., Adewopo O.A. (2016). Grain iron and zinc concentrations and their relationship with selected agronomic traits in early and extra-early maize. J. Crop Improv..

[56] Kihara J., Sileshi G.W., Bolo P., Mutambu D., Senthilkumar K., Sila A., Devkota M., Saito K. (2024). Maize-grain zinc and iron concentrations as influenced by agronomic management and biophysical factors: A meta-analysis. Food Secur..

[57] Mageto E.K., Lee M., Dhliwayo T., Palacios-Rojas N., San Vicente F., Burgueño J., Hallauer A.R. (2020). An evaluation of kernel zinc in hybrids of elite quality protein maize (QPM) and non-QPM inbred lines adapted to the tropics based on a mating design. Agronomy.

