# Supplementary material for: Unveiling the Physical and Nutritional Profiling of Grains in Maize Landraces and Hybrids
Source: Foods. 2026 Aug 21;15(16):2933. doi: 10.3390/foods15162933 (PMC13512079; doi:10.3390/foods15162933)
Supplement: Supplementary file 1 [file foods-15-02933-s001.zip › foods-4402314-supplementary.pdf]

Sample code	Type	Origin	Key nutritional trait	Value	Unit
HIDA 131	Landrace	CH-1994-U--172	Protein	15.76	%
JALI300	Landrace	TL-2000-A-1903-28	Protein	15.57	%
PBS-002	Landrace	Sinaloa	Ether extract	11.80	%
CON-101	Landrace	Sinaloa	Fe	53.31	mg kg^{-1}
CR. PISCORUNTO II	Landrace	CQ-1995-U--241	Zn	45.22	mg kg^{-1}
URUG,228A-#	Landrace	BA13-13BQ2004-32	Total carotenoids	51.23	µg g^{-1}
CHLHW12013	Hybrid	BA13-2113-7/2113-8	Protein	15.30	%
CLTHY11002	Hybrid	Yucatán, 2016	Starch	76.81	%
CHLHY09002	Hybrid	Valles Altos, 2016	Starch	75.64	%
CLTHY010	Hybrid	04-14MASTTHY-1	Total carotenoids	32.24	µg g^{-1}
CLTHW15001	Hybrid	Santa Fe, Hopelchén	Zn	30.03	mg kg^{-1}

Type	Sample code*	Key nutritional trait	Value	Unit
Landrace	HIDA 131	Protein	15.76	%
Landrace	JALI300	Protein	15.57	%
Landrace	PBS-002	Ether extract	11.80	%
Landrace	CON-101	Fe	53.31	mg kg^{-1}
Landrace	CR. PISCORUNTO II	Zn	45.22	mg kg^{-1}
Landrace	URUG,228A	Total carotenoids	51.23	µg g^{-1}
Hybrid	CHLHW12013	Protein	15.30	%
Hybrid	CLTHY11002	Starch	76.81	%
Hybrid	CHLHY09002	Starch	75.64	%
Hybrid	CLTHY010	Total carotenoids	32.24	µg g^{-1}
Hybrid	CLTHW15001	Zn	30.03	mg kg^{-1}

*Germplasm available from CIMMYT genebank
